# Assessment of methadone maintenance therapy implementation and perceived barriers to treatment participation: A mixed-methods study

**DOI:** 10.1097/MD.0000000000050507

**Published:** 2026-09-11

**Authors:** Dang Duc Nhu, Nguyen Ngoc Nghia, Nguyen Anh Tuan, Truong Ky Phong, Le Xuan Nam, Nguyen Thanh Trung, Phung Lam Toi, Nguyen Huynh Phuong Anh, Tran Thi Ly, Nguyen Trong Khoa, Duong Huy Luong, Tran Quoc Thang, Tran Vuong The Vinh, Luong Thi Thu Ha, La Ngoc Quang

**Affiliations:** a Faculty of Public Health, University of Medicine and Pharmacy, Vietnam National University at Hanoi, Hanoi, Vietnam; b Ha Dong Health Center, Hanoi, Vietnam; c Department of Medical Service Administration, Ministry of Health, Hanoi, Vietnam; d Faculty of Public Health, Phenikaa School of Medicine and Pharmacy, Phenikaa University, Hanoi, Vietnam; e Department of Surgery, Hai Phong University of Medicine and Pharmacy, Hai Phong, Vietnam; f Thang Long Institute of Medical and Pharmaceutical Research, Hanoi, Vietnam; g Department of Epidemiology, Faculty of Fundamental Sciences, Hanoi University of Public Health, Hanoi, Vietnam.

**Keywords:** healthcare service delivery, implementation, methadone maintenance therapy, mixed-methods study, opioid dependence, treatment participation

## Abstract

Methadone maintenance therapy (MMT) effectiveness depends not only on the pharmacological effects of methadone but also on the quality of program implementation and service delivery. This study aimed to assess MMT implementation according to national guidelines and explore perceived barriers to treatment participation. A mixed-methods cross-sectional study was conducted from October 2022 to October 2023. The quantitative component included all eligible patients receiving MMT during the study period (n = 230), and implementation indicators included dose initiation, dose adjustment, toxicity assessment, monitoring practices, adverse-effect management, and management of missed doses. The qualitative component involved semi-structured interviews with 5 patients, two healthcare workers, and 2 facility manager. Quantitative data were analyzed descriptively, while qualitative data were analyzed using thematic analysis. The mean treatment duration was 52.2 ± 45.2 months. Overall, MMT implementation showed high compliance with national guidelines. Appropriate initial dosing, daily clinical assessment, dose adjustment, adverse-effect management, and management of missed doses exceeded 95% compliance. Toxicity assessment before dose escalation was documented in 71.7% of cases, representing the main implementation gap. No overdose or incorrect medication administration was identified. Qualitative findings revealed policy-related, facility-related, and patient-related barriers to treatment participation, including daily medication pick-up requirements, fixed operating hours, staff shortages, infrastructure limitations, employment obligations, and ongoing illicit substance use. MMT was implemented largely in accordance with national guidelines. Improving toxicity monitoring, service accessibility, staffing capacity, and operational procedures may further enhance the quality and accessibility of MMT services.

## 1. Introduction

Drug use remains a major global public health challenge, contributing substantially to morbidity, mortality, and social burdens worldwide. According to the United Nations Office on Drugs and Crime, approximately 284 million people aged 15 to 64 years used drugs at least once in 2020, of whom 38.6 million suffered from drug use disorders. Drug use was responsible for an estimated 494,000 deaths and 30.9 million years of healthy life lost globally in 2019.^[1,2]^ In Vietnam, approximately 235,000 people were reported to use illicit drugs as of December 2020.^[3]^

Opioids are among the most commonly used illicit substances worldwide, with an estimated 61 million users globally.^[4]^ Opioid dependence is associated with a wide range of adverse health outcomes, including overdose, infectious diseases, psychiatric disorders, and premature mortality. Methadone maintenance therapy (MMT) has been widely recognized as an effective and evidence-based treatment for opioid dependence. Previous studies have demonstrated that MMT reduces illicit opioid use, improves physical and mental health, decreases criminal behaviors, and enhances social functioning and quality of life.^[5-7]^

The effectiveness of MMT depends not only on the pharmacological properties of methadone but also on the quality of program implementation at treatment facilities. Appropriate dose initiation, dose adjustment, patient monitoring, management of adverse effects, and handling of missed doses are essential components of treatment delivery. In addition, operational factors such as medication dispensing procedures, clinic accessibility, staffing capacity, and service organization may influence patients’ participation in treatment programs and their ability to maintain regular attendance.^[8]^ Although the effectiveness of MMT has been extensively studied, evidence regarding the implementation of MMT programs in routine clinical practice remains limited, particularly in low- and middle-income countries. Furthermore, few studies have simultaneously evaluated compliance with treatment guidelines and explored barriers to treatment participation from the perspectives of both patients and healthcare providers.

Stigma may further influence whether people with opioid use disorder initiate and remain engaged in opioid agonist treatment. People receiving methadone treatment may experience anticipated, internalized, or enacted stigma related both to their history of substance use and to being identifiable as recipients of opioid agonist treatment. Such experiences may discourage treatment seeking, create concerns regarding disclosure and confidentiality, contribute to conflicts in social relationships, and increase reluctance to initiate, access, or continue methadone treatment. Evidence from Vietnam also indicates that drug-related stigma is associated with reduced access to healthcare and that patients’ experiences of stigma may differ according to the service-delivery model. Service organization, healthcare-provider attitudes, privacy protections, and the extent to which methadone treatment is integrated into broader healthcare services may therefore influence treatment accessibility and retention.^[9-12]^

Therefore, this study aimed to assess the implementation of MMT according to national guidelines and to explore perceived barriers to treatment participation among patients and healthcare workers at a methadone treatment facility in Hanoi, Vietnam.

## 2. Materials and methods

### 2.1. Study design, setting and participants

This descriptive cross-sectional mixed-methods study was conducted at the Ha Dong Methadone Treatment Facility in Hanoi, Vietnam, from October 2022 to October 2023. The study was approved by the Institutional Review Board of the University of Public Health (IRB No. 301/2023/YTCC-HD3). Participation in the qualitative component was voluntary, and informed consent was obtained from all interview participants. During the study period, the facility provided MMT in accordance with Decision No. 3140/QD-BYT, issued by the Ministry of Health of Vietnam on August 30, 2010, which constituted the principal national clinical guideline applicable to MMT delivery. Organizational procedures for methadone dispensing facilities were additionally governed by Decision No. 3509/QD-BYT, dated August 21, 2015.^[13,14]^

The study comprised 2 components. The quantitative component involved a retrospective review of medical records to assess the implementation of MMT procedures and concordance with national treatment guidelines. All patients receiving MMT at the facility during the study period were considered eligible. Records lacking information required for the implementation assessment were excluded.

### 2.2. Data collection

Data were extracted from medical records using a standardized data extraction form developed by the research team. Information collected included demographic characteristics, treatment duration, methadone dosing practices, dose adjustments, monitoring activities, adverse effect management, and management of missed doses. To ensure data quality, completed extraction forms were reviewed by a senior investigator before data entry.

### 2.3. Sample size and sampling

The quantitative component included all eligible patients receiving treatment at the facility during the study period. A total of 230 patients met the inclusion criteria and were included in the analysis. Because all available eligible patients were recruited, the study effectively represented a census of the treatment population at the facility rather than a sampled subset.

### 2.4. Study variables

The primary quantitative outcomes were indicators reflecting implementation of MMT according to national guidelines, including appropriate dose initiation, appropriate dose adjustment, toxicity assessment before dose escalation, monitoring practices, adverse effect management, and management of missed doses.

Additional descriptive variables included age, sex, educational level, marital status, treatment duration, and comorbid conditions.

### 2.5. Assessment of MMT implementation

The implementation assessment was based on Decision No. 3140/QD-BYT of the Ministry of Health of Vietnam, the national methadone treatment guideline applicable during the study period.^[13]^ The guideline describes voluntary, long-term methadone treatment combined with psychosocial and medical support and specifies procedures for induction, dose adjustment, maintenance treatment, clinical monitoring, toxicity assessment, management of adverse effects, and management of treatment interruptions.

The following domains were evaluated.

#### 2.5.1. Dose initiation

Compliance with national recommendations regarding initial methadone dosing (15–30 mg/d) was assessed.

#### 2.5.2. Dose adjustment

The recommended initial methadone dose was 15 to 30 mg/d, with an average starting dose of approximately 20 mg/d and additional caution when initiating treatment at 25 to 30 mg/d. During the induction phase, patients were to be assessed daily before medication administration. When withdrawal symptoms, craving, or continued opioid use persisted without evidence of toxicity, the dose could generally be increased by 5 to 10 mg after 3 to 5 days, with the total increase not exceeding 20 mg/wk. From the dose-adjustment phase beginning approximately in the third week, the dose could be increased by 5 to 15 mg after 3 to 5 days, with a maximum total increase of 30 mg/wk. Clinicians were required to assess patients for intoxication or other signs of methadone toxicity before increasing the dose. The usual recommended maintenance-dose range was 60 to 120 mg/d, although the effective dose was individualized according to clinical response.

#### 2.5.3. Monitoring

Documentation of patient monitoring, including withdrawal symptoms, risk behaviors, disease progression, and urine testing practices, was assessed.

#### 2.5.4. Management of adverse effects

Records were reviewed to determine whether adverse effects were appropriately documented and managed according to clinical guidelines.

#### 2.5.5. Management of missed doses

Management of missed doses was evaluated according to national recommendations:

- Missed doses for 1 to 3 days: continuation of the previous methadone dose.
- Missed doses for 4 to 5 consecutive days: reassessment of opioid tolerance and administration of approximately half of the previous dose, followed by dose re-titration as needed.
- Missed doses for more than 5 consecutive days: reinitiation of methadone treatment from the starting dose.

#### 2.5.6. Qualitative component

A qualitative exploratory approach was used to identify perceived barriers to participation in the MMT program. Purposive sampling was employed to recruit individuals with direct experience of the treatment process. Participants included 5 patients receiving methadone treatment, 2 healthcare workers directly involved in service delivery (one physician and one nurse), and the facility manager. Semi-structured, in-depth interviews were conducted using an interview guide developed by the research team. Interview topics included treatment procedures, service accessibility, operational challenges, healthcare staffing, facility conditions, and factors influencing participation in the MMT program.

Audio-recorded interviews were transcribed verbatim and analyzed using thematic analysis. To enhance trustworthiness, 2 researchers independently coded transcripts, and discrepancies were resolved through discussion. Representative quotations were used to support identified themes.

### 2.6. Statistical analysis

Quantitative data were analyzed using SPSS version 22.0 (IBM Corp., Armonk). Continuous variables were presented as means and standard deviations or medians and interquartile ranges, as appropriate. Categorical variables were summarized using frequencies and percentages. Because the primary objective was to describe MMT implementation practices rather than test hypotheses regarding treatment efficacy, analyses were mainly descriptive.

## 3. Results

### 3.1. Participant characteristics

A total of 230 patients receiving MMT were included in the study. The mean age was 45.5 years (range: 26–83 years), and the average treatment duration was 52.2 ± 45.2 months. Most participants were male (97.8%), married or living with a partner (73.5%), and had completed upper secondary education (42.6%). Nearly all patients were in the maintenance phase of treatment (99.1%). The prevalence of comorbid conditions included hepatitis C (44.4%), hepatitis B (7.8%), and human immunodeficiency virus infection (6.5%; Table 1).

**Table 1:** Characteristics of patients receiving methadone maintenance therapy (n = 230).

Characteristics	Number	%
Mean age (yr)	45.5 (range: 26–83)	
Gender		
Male	225	97.8
Female	5	2.2
Marital status		
Single	53	23.0
Married/partnered	169	73.5
Separated/divorced/widowed	8	3.5
Education level		
Primary or below	55	23.9
Lower secondary	72	31.3
Upper secondary	98	42.6
College/graduate	5	2.2
Treatment phase		
Dose adjustment	2	0.9
Maintenance	228	99.1
Treatment duration (mo)	52.2 ± 45.2	
Comorbidities		
Hepatitis B	18	7.8
Hepatitis C	128	44.4
HIV	15	6.5

HIV = human immunodeficiency virus.

### 3.2. Implementation of methadone maintenance therapy according to national guidelines

Overall, implementation of MMT at the study facility demonstrated a high level of compliance with national treatment guidelines. Appropriate initial dosing, daily monitoring during dose titration, dose adjustment procedures, adverse-effect management, and management of missed doses were documented in more than 95% of patients. A comparison between guideline requirements and observed implementation indicators is presented in Table S2, Supplemental Digital Content 1. No cases of overdose or incorrect medication administration were identified during the study period.

The mean initial methadone dose was 20.7 ± 10.1 mg/d. Almost all patients (99.6%) underwent daily clinical assessment during the dose titration period, and dose titration was performed according to national guidelines. Most patients achieved dose stabilization within 3 weeks after treatment initiation.

The most common dose adjustment practice was an increase of 10 mg per adjustment (99.1%). The time from dose escalation to stabilization was approximately 30 days in most patients (94.3%). Prior to dose escalation, toxicity assessment was documented in 71.7% of patients. The mean methadone dose at the beginning of the maintenance phase was 32.3 mg/d, while the current mean maintenance dose was 59.2 ± 28.2 mg/d. No dose reductions were recorded during the study period.

Adverse effects were appropriately documented and managed in accordance with the study-period national guideline in 229 of 230 patients (99.6%). The value 229 represents the number meeting this implementation indicator and not a reduction in the overall study sample. The lowest-performing implementation indicator was toxicity assessment before dose escalation, which was documented in 71.7% of cases (Table 2).

**Table 2:** Implementation indicators for methadone maintenance therapy according to the national guideline applicable during the study period.

Implementation indicator	Number	%
Appropriate initial methadone dose	229	99.6
Daily clinical assessment during dose titration	229	99.6
Dose titration performed according to guidelines	229	99.6
Dose adjustment performed according to guidelines	225	97.8
Toxicity assessment before dose escalation	165	71.7
Adverse effects appropriately documented and managed	229	99.6
Missed doses managed according to guidelines (n = 219)	219	100.0
Overdose cases	0	0
Incorrect medication administration	0	0
Mean methadone dose at maintenance initiation (mg/d)	32.3	
Current methadone dose (mg/d)	59.2 ± 28.2	

Unless otherwise specified, percentages were calculated using all 230 included patients as the denominator. For the missed-dose management indicator, the denominator was the 219 patients who experienced at least one documented missed-dose episode; all 219 patients were managed in accordance with the guideline.

### 3.3. Management of missed doses

A total of 219 patients (95.2%) experienced at least one missed-dose episode during treatment. Among all 230 patients, 206 (89.6%) had interruptions lasting 1 to 3 days, 13 (5.7%) had interruptions lasting 4 to 5 days, and 11 (4.8%) had no missed doses. All 219 patients with at least one documented missed-dose episode were managed according to national recommendations (Table 3).

**Table 3:** Characteristics of missed-dose episodes during treatment.

	Number	%
Duration of missed doses		
1–3 d	206	89.6
4–5 d	13	5.7
No missed doses	11	4.8
Treatment stage when missed doses occurred		
Titration phase	106	46.1
Dose adjustment phase	88	38.3
Maintenance phase	25	10.9
Managed according to guideline	219	100.0

### 3.4. Perceived barriers to treatment participation

Qualitative interviews identified 3 major categories of barriers to treatment participation: policy-related barriers, facility-related barriers, and patient-related barriers (Table 4).

**Table 4:** Summary of perceived barriers to treatment participation.

Main theme	Subtheme	Reported impact
Policy-related barriers	Daily medication pick-up requirement	Interfered with employment and daily activities
	Treatment fees	Initial financial burden, although generally acceptable
Facility-related barriers	Fixed operating hours	Difficulty attending treatment before work
	Manual queue management	Delays and inconvenience during medication collection
	Facility location	Reduced accessibility for some patients
	Inadequate infrastructure	Affected staff working conditions and record storage
	Staff shortages	Reduced counseling opportunities and patient interaction
Patient-related barriers	Employment obligations	Competing work demands
	Ongoing substance use	Reduced engagement in treatment
	Early treatment stage	Increased monitoring and clinic visits required

#### 3.4.1. Policy-related barriers

Daily medication pick-up was the most frequently reported policy-related challenge. Both patients and healthcare providers indicated that mandatory daily attendance interfered with employment and routine activities. Although treatment fees had shifted from being fully subsidized to requiring patient contributions, most participants considered the current monthly fee affordable and acceptable.

“The daily medication collection does impact the patient’s work, but it’s a mandatory requirement.” (Physician)

#### 3.4.2. Facility-related barriers

Several operational barriers were identified at the facility level. Patients reported difficulties related to fixed operating hours, particularly the 7:30 am medication schedule, which conflicted with work obligations. Manual queue management occasionally resulted in delays and dissatisfaction. Participants also highlighted the inconvenient location of the treatment facility, inadequate infrastructure, and shortages of healthcare personnel. Healthcare workers noted that staffing constraints limited their ability to provide counseling and patient follow-up.

“If it’s set at 7:30 AM, it’s a bit late for patients who need to go to work early.” (Physician)

#### 3.4.3. Patient-related barriers

Patient-level barriers included employment obligations, ongoing illicit drug use, and challenges during the early stages of treatment. Healthcare providers reported that some patients continued using illicit substances while receiving methadone therapy, which complicated treatment management. The dose titration period was also perceived as particularly demanding because of the need for frequent assessments and consultations.

“Some patients request a dose reduction... because they are using other drugs outside.” (Physician)

Additional representative quotations supporting each theme are presented in Table S1, Supplemental Digital Content 2.

## 4. Discussion

The study demonstrated a high level of compliance with most implementation indicators, including appropriate dose initiation, dose titration, dose adjustment, adverse-effect management, and management of missed doses. Qualitative findings further identified policy-related, facility-related, and patient-related barriers that may affect participation in MMT programs.

A major finding of this study was the high overall compliance of MMT implementation with national treatment guidelines. As summarized in Table S2, Supplemental Digital Content 1, most implementation indicators exceeded 95% compliance, including appropriate dose initiation, daily assessment during dose titration, dose adjustment procedures, adverse-effect management, and management of missed doses. These findings indicate that the study facility had established robust clinical and operational processes for delivering MMT in accordance with Ministry of Health recommendations. However, toxicity assessment before dose escalation was documented in only 71.7% (n = 165/230) of patients, representing the largest implementation gap identified in the present study. Although this lower rate may partly reflect incomplete documentation in medical records, it highlights an important area for quality improvement. Ensuring consistent toxicity assessment prior to dose escalation is essential for optimizing treatment safety and maintaining adherence to evidence-based methadone treatment standards. Given the well-established benefits of MMT in reducing illicit opioid use, improving health outcomes, and enhancing social functioning among individuals with opioid dependence, maintaining high implementation quality is critical for maximizing the effectiveness of treatment programs.^[15-18]^

The Vietnamese recommendations applicable during the study period were broadly consistent with World Health Organization guidance on several core clinical components (Table S3, Supplemental Digital Content 3). Both recommended individualized low-dose induction, an initial dose not exceeding 30 mg, careful assessment for sedation or toxicity during dose escalation, maintenance doses commonly in the range of 60 to 120 mg/d, clinical monitoring and drug testing, and the provision of psychosocial support alongside pharmacological treatment. However, the service-delivery models differed in flexibility. The Vietnamese system at the time of this study generally required patients to attend a treatment facility for directly supervised daily dosing. World Health Organization recommends direct supervision during the early phase but supports individualized take-home dosing for stable patients when the benefits of reducing attendance exceed the risks of diversion. This difference is particularly relevant to the employment-related and operating-hour barriers reported by participants. The study findings therefore reflect both the high clinical standardization provided by the national guideline and the accessibility burden associated with its implementation model.^[13,14,17,18]^

The current mean maintenance methadone dose was 59.2 ± 28.2 mg/d, with considerable between-patient variation. Beyond dose optimization, sustained engagement in treatment is important for improving long-term outcomes. Huang et al reported that a longer cumulative duration of methadone maintenance treatment was associated with lower all-cause mortality in a nationwide retrospective cohort study.^[19]^ This finding further emphasizes the importance of addressing policy-, facility-, and patient-related barriers that may disrupt continued participation in MMT.

The qualitative findings provided important insights into barriers affecting treatment participation. At the policy level, the requirement for daily medication pick-up was perceived as a major challenge, particularly among employed patients. Similar findings have been reported in both international and Vietnamese studies, where frequent attendance requirements were associated with difficulties maintaining employment and regular participation in treatment programs. Previous studies have also reported substantial levels of treatment nonadherence or treatment discontinuation among MMT patients, ranging from 38.2% to 73.4% depending on the setting. Although the present study did not evaluate adherence outcomes, these findings suggest that operational barriers may contribute to difficulties in maintaining long-term engagement with treatment services.^[8,20-23]^

Several facility-related barriers were identified, including fixed operating hours, manual queue management, staff shortages, and inadequate infrastructure. Previous studies have demonstrated that organizational and structural factors play an important role in access to addiction treatment services and long-term retention in care. In the present study, both patients and healthcare providers highlighted the impact of operating hours and workforce shortages on service accessibility. Limited staffing may reduce opportunities for counseling, patient education, and individualized follow-up, all of which are important components of comprehensive MMT delivery.^[24-28]^

Stigma should also be considered when interpreting treatment accessibility. Previous Vietnamese studies have documented addiction-related blame, shame, concerns about disclosure, community discrimination, and fear of being identified as an MMT patient. The national guidance relevant to the study period addressed stigma indirectly by defining opioid dependence as a chronic, relapsing medical condition, emphasizing voluntary treatment, incorporating psychosocial support, and requiring confidentiality of patient information. These provisions may reduce moral judgment and involuntary disclosure; however, the guidance did not specify a dedicated anti-stigma intervention or measurable anti-stigma implementation indicators.^[9,12-14]^

At the study facility, counseling services and generally positive perceptions of healthcare staff may have supported a respectful treatment environment. Nevertheless, staff shortages reduced opportunities for counseling and individualized interaction. Daily attendance, fixed dispensing hours, queuing, and attendance at an identifiable methadone facility could also increase the visibility of patients’ treatment status and thereby contribute to anticipated stigma or confidentiality concerns. These mechanisms should be interpreted cautiously because stigma was not directly or systematically measured in this study. Consequently, we cannot determine whether stigma affected attendance, missed doses, retention, or concordance with the clinical guideline.

Patient-related barriers included employment obligations, ongoing illicit substance use, and challenges encountered during the early stages of treatment. Continued drug use during MMT remains a recognized challenge in opioid dependence treatment and may complicate dose management, monitoring, and patient engagement. Healthcare providers in the present study reported that some patients continued using illicit substances despite receiving methadone treatment, highlighting the importance of ongoing counseling and behavioral support alongside pharmacological treatment.^[23,29,30]^

The findings of this study have several practical implications. Extending clinic operating hours, implementing electronic queue-management systems, strengthening healthcare workforce capacity, and improving facility infrastructure may help reduce barriers to treatment participation. In addition, reinforcing documentation practices and toxicity monitoring before dose escalation could further improve compliance with national treatment guidelines and enhance the quality of MMT service delivery.

This study has several strengths. By combining quantitative assessment of implementation indicators with qualitative exploration of patient and provider experiences, the study provides a comprehensive evaluation of MMT service delivery in a real-world clinical setting. Furthermore, all eligible patients attending the facility during the study period were included in the quantitative component, reducing the risk of selection bias.

Several limitations should also be acknowledged. First, the study was conducted at a single treatment facility, which may limit the generalizability of the findings to other settings. Second, the quantitative assessment relied on retrospective medical records and was therefore dependent on the completeness and accuracy of documentation. Third, the qualitative component involved a relatively small number of participants, although sampling included both patients and healthcare providers with direct experience of the treatment process. Stigma was not assessed using a validated quantitative instrument or included as a dedicated prespecified qualitative domain. The study therefore could not estimate anticipated, internalized, or enacted stigma or determine whether stigma influenced treatment initiation, attendance, missed doses, retention, or implementation outcomes. Moreover, because the study included patients who were already receiving MMT, it did not capture treatment-eligible individuals who may have avoided enrollment because of stigma, concerns about disclosure, or distrust of treatment services. Future multicenter prospective studies should include both current patients and people who are eligible for but not enrolled in MMT, apply validated stigma measures, examine the privacy and integration of treatment facilities, and assess associations between stigma and treatment initiation, adherence, retention, and guideline implementation. Finally, because the study was descriptive in nature, it was not designed to evaluate treatment efficacy or establish causal relationships between identified barriers and treatment outcomes.

## 5. Conclusion

MMT at the study facility was implemented largely in accordance with national guidelines, with high compliance across most implementation indicators. Toxicity assessment before dose escalation was the main implementation gap identified. Perceived barriers to treatment participation included daily medication pick-up requirements, fixed operating hours, staff shortages, infrastructure limitations, employment obligations, and ongoing illicit substance use. Addressing these operational barriers and strengthening monitoring procedures may further improve the quality and accessibility of MMT services.

## Author contributions

**Conceptualization:** Dang Duc Nhu.

**Funding acquisition:** Duong Huy Luong.

**Investigation:** Dang Duc Nhu, Nguyen Anh Tuan, Le Xuan Nam, Nguyen Ngoc Nghia, Phung Lam Toi, Tran Thi Ly, Nguyen Trong Khoa, Duong Huy Luong, Tran Quoc Thang.

**Methodology:** Dang Duc Nhu, Nguyen Anh Tuan, Truong Ky Phong, Le Xuan Nam, Nguyen Ngoc Nghia, Phung Lam Toi, Tran Thi Ly, Nguyen Trong Khoa, Tran Vuong The Vinh, La Ngoc Quang.

**Project administration:** Dang Duc Nhu, Nguyen Anh Tuan, Truong Ky Phong, Le Xuan Nam, Nguyen Thanh Trung, Nguyen Ngoc Nghia, Phung Lam Toi, Nguyen Huynh Phuong Anh, Nguyen Trong Khoa, Tran Quoc Thang, Tran Vuong The Vinh, Luong Thi Thu Ha, La Ngoc Quang.

**Resources:** Dang Duc Nhu, Nguyen Anh Tuan, Truong Ky Phong, Le Xuan Nam, Nguyen Thanh Trung, Nguyen Ngoc Nghia, Phung Lam Toi, Nguyen Huynh Phuong Anh, Tran Thi Ly, Nguyen Trong Khoa, Duong Huy Luong, Tran Quoc Thang, Luong Thi Thu Ha.

**Software:** Nguyen Anh Tuan, Le Xuan Nam, Nguyen Thanh Trung, Nguyen Ngoc Nghia, Phung Lam Toi, Nguyen Huynh Phuong Anh, Tran Thi Ly, Nguyen Trong Khoa, Tran Quoc Thang, Tran Vuong The Vinh, Luong Thi Thu Ha.

**Supervision:** Dang Duc Nhu.

**Validation:** Dang Duc Nhu, Nguyen Anh Tuan, Truong Ky Phong, Le Xuan Nam, Nguyen Thanh Trung, Nguyen Huynh Phuong Anh, Tran Thi Ly, Tran Vuong The Vinh, Luong Thi Thu Ha, La Ngoc Quang.

**Visualization:** Dang Duc Nhu, Nguyen Anh Tuan, Truong Ky Phong, Le Xuan Nam, Nguyen Ngoc Nghia, Phung Lam Toi, Tran Thi Ly, Nguyen Trong Khoa, Duong Huy Luong, Tran Quoc Thang, La Ngoc Quang.

**Writing – original draft:** Dang Duc Nhu, Nguyen Anh Tuan, Truong Ky Phong, Le Xuan Nam, Nguyen Thanh Trung, Nguyen Ngoc Nghia, Phung Lam Toi, Nguyen Huynh Phuong Anh, Tran Thi Ly, Nguyen Trong Khoa, Duong Huy Luong, Tran Quoc Thang, Tran Vuong The Vinh, Luong Thi Thu Ha, La Ngoc Quang.

**Writing – review & editing:** Dang Duc Nhu, Nguyen Anh Tuan, Truong Ky Phong, Le Xuan Nam, Nguyen Thanh Trung, Nguyen Ngoc Nghia, Phung Lam Toi, Nguyen Huynh Phuong Anh, Tran Thi Ly, Nguyen Trong Khoa, Duong Huy Luong, Tran Quoc Thang, Tran Vuong The Vinh, Luong Thi Thu Ha, La Ngoc Quang.






