# Supplementary material for: Assessment of methadone maintenance therapy implementation and perceived barriers to treatment participation: A mixed-methods study
Source: Medicine (Baltimore). 2026 Sep 11;105(37):e50507. doi: 10.1097/MD.0000000000050507 (PMC13574426; doi:10.1097/MD.0000000000050507)
Supplement: Supplementary file 1 [file medi-105-e50507-s001.docx]

| Domain | Guideline requirement | Observed compliance |
| --- | --- | --- |
| Initial dosing | 15–30 mg/day | 99.6% |
| Daily assessment during titration | Required | 99.6% |
| Dose adjustment procedure | Required | 97.8% |
| Toxicity assessment before escalation | Required | 71.7% |
| Adverse-effect management | Required | 99.6% |
| Missed-dose management | Required | 100.0% |
|  |  |  |

**Supplementary Table 2. Comparison between national guideline requirements and observed implementation indicators**
