# Supplementary material for: Assessment of methadone maintenance therapy implementation and perceived barriers to treatment participation: A mixed-methods study
Source: Medicine (Baltimore). 2026 Sep 11;105(37):e50507. doi: 10.1097/MD.0000000000050507 (PMC13574426; doi:10.1097/MD.0000000000050507)
Supplement: Supplementary file 2 [file medi-105-e50507-s002.docx]

**Supplementary Table 1. Representative quotations supporting themes and sub-themes related to barriers to treatment participation in methadone maintenance therapy**

| Main theme | Sub-theme | Participant | Representative quotation |
| --- | --- | --- | --- |
| Policy-related barriers | Daily medication pick-up | Physician | “The daily medication collection does impact the patient's work, but it's a mandatory requirement.” |
|  | Treatment fees | Facility manager | “It used to be free, funded by the Vietnam and the U.S. governments, but now patients have to contribute. However, I find the patient’s contribution to be small compared to the real cost, so there's nothing concerning, and patients haven't complained.” |
|  | Treatment fees | Nurse | “At first, collecting fees was a bit difficult because patients had to pay, but now that we are familiar with them, it's not challenging anymore.” |
|  | Treatment fees | Patient participant 1 | “The actual cost is high, but the government covers most of it; we only pay a few hundred thousand Vietnam dong.” |
|  | Treatment fees | Patient participant 5 | “In my opinion, we take the medication for our own benefit. A fee of 300,000 VND per month is reasonable and does not affect us much.” |
|  | Treatment regulations | Physician | “The regulations are reasonable. For example, the dose titration period is well-timed and does not need to be longer or shorter.” |
|  | Continued illicit drug use under current regulations | Physician | “Patients can still take methadone, but some continue using heroin. At our facility, we can only advise them to voluntarily comply, without any enforcement or checks.” |
|  | Operating hours | Patient participant 5 | “I suggest that the center allows earlier medication. We have to work and taking medication at 7:30 AM is difficult because most of us need to go to work.” |
|  | Operating hours | Physician | “If it's set at 7:30 AM, it’s a bit late for patients who need to go to work early. It also helps reduce unnecessary crowds at the medication point.” |
|  | Queue management | Patient participant 5 | “Even if I put my card first, the order gets messed up. There is no staff to manage it, so we do it ourselves.” |
|  | Facility location | Physician | “The location here is quite populated, and having a separate area would often be better for the patients and the community.” |
|  | Facility location | Patient participant 3 | “I find it inconvenient to get here, but I still make the effort.” |
|  | Infrastructure | Nurse | “The facilities are still lacking, with mold and wet cabinets, which also makes the medical records moldy.” |
|  | Infrastructure | Physician | “The facilities here are really in a bad state, but there have not been any repairs.” |
|  | Infrastructure (positive perception) | Patient participant 1 | “There is already water, space, and parking available, so there is nothing to improve.” |
|  | Staff shortages | Facility manager | “We are short on healthcare staff. For example, we need three pharmacists, but we only have two here.” |
|  | Staff shortages | Facility manager | “Counseling requires two people, but we only have one, so they cannot provide thorough counseling.” |
|  | Limited interaction with healthcare staff | Patient participant 5 | “Since then, I have not spoken to him unless I requested a dose reduction.” |
|  | Positive healthcare staff attitude | Patient participant 2 | “I decided to come here because the treatment method and patient care are relatively good.” |
| Patient-related barriers | Financial concerns | Nurse | “At first, it was a bit difficult because patients had to pay.” |
|  | Employment obligations | Patient participant 5 | “We have to work; taking medication at 7:30 AM is difficult.” |
|  | Ongoing substance use | Physician | “Some patients request a dose reduction. It is not because they cannot handle the side effects of the medication, but because they are using other drugs outside.” |
|  | Early treatment stage | Facility manager | “New patients are usually more challenging because they need continuous examinations and consultations to reach the optimal dose.” |

MMT, methadone maintenance therapy.
