# Supplementary material for: Assessment of methadone maintenance therapy implementation and perceived barriers to treatment participation: A mixed-methods study
Source: Medicine (Baltimore). 2026 Sep 11;105(37):e50507. doi: 10.1097/MD.0000000000050507 (PMC13574426; doi:10.1097/MD.0000000000050507)
Supplement: Supplementary file 3 [file medi-105-e50507-s003.docx]

**Supplementary Table 3. Comparison of the Vietnamese national guideline for methadone maintenance treatment applicable during the study period with World Health Organization recommendations for opioid agonist treatment**

| Domain | Vietnam guideline applicable in 2022–2023 | WHO recommendations | Interpretation |
| --- | --- | --- | --- |
| Treatment approach | Voluntary, long-term methadone treatment with psychosocial and medical support | Opioid agonist maintenance treatment, usually methadone or buprenorphine, with psychosocial support | Broad concordance |
| Initial dose | 15–30 mg/day; average approximately 20 mg | Generally ≤20 mg; never >30 mg | Broad concordance |
| Dose escalation | 5–10 mg every 3–5 days during induction; ≤20 mg/week; later 5–15 mg every 3–5 days; ≤30 mg/week | Rapid but cautious individualized titration with monitoring for withdrawal and sedation | Vietnam provides more prescriptive intervals |
| Maintenance dose | Usually 60–120 mg/day | Usually 60–120 mg/day | Concordant |
| Supervision | Daily supervised administration was standard during the study | Supervised initially; take-home doses may be permitted for stable patients after individualized risk–benefit assessment | WHO model is more flexible |
| Monitoring | Withdrawal, toxicity, urine testing, adverse effects and missed doses | Clinical assessment, drug testing, treatment records and safety monitoring | Broad concordance |
| Psychosocial support | Required as part of treatment | Routinely offered with pharmacological treatment | Concordant |
| Confidentiality and stigma | Confidentiality required under related regulations; chronic-disease and voluntary-treatment framing; no dedicated stigma indicators | Explicit confidentiality, ethical and person-centered care, reduced access restrictions and non-discriminatory services | International standards are more explicit |

**Sources:** Vietnam Ministry of Health Decisions No. 3140/QD-BYT and No. 3509/QD-BYT [13,14]; World Health Organization and United Nations Office on Drugs and Crime guidance [17,18]
